# Heterozygous *HTRA1* missense mutation in CADASIL-like family disease

**DOI:** 10.1590/1414-431X20176632

**Published:** 2018-03-15

**Authors:** Xiaowei Wu, Changxin Li, Jinming Mao, Ling Li, Yan Liu, Yao Hou

**Affiliations:** Department of Neurology, the First Hospital of Shanxi Medical University, Taiyuan, China

**Keywords:** HTRA1, CADASIL-like, SNP, Cerebral small vessel disease

## Abstract

The aim of this study was to find related pathogenic genes in cerebral autosomal dominant arteriopathy with subcortical infarcts and leukoencephalopathy in (CADASIL)-like patients. The direct sequencing and high-throughput multiplex polymerase chain reaction (PCR) was performed to screen for related genes. The clinical and imaging data of a CADASIL-like patient (the pro-band) and his family members were collected. At first, the known hereditary cerebral vascular genes of the pro-band were screened with direct sequencing to find candidate gene mutations. High-throughput multiplex PCR was then used to analyze the single nucleotide polymorphism of the candidate gene in the family members. The results showed that there was missense mutation of the high temperature requirement protease A1 (*HTRA1*) gene in the pro-band, which may be a pathogenic factor according to the biological software analysis. The following SNP results revealed that the other family members also had the *HTRA1* gene mutation. Thus, the CADASIL-like family disease may be caused by heterozygous *HTRA1* gene mutation, which leads to autosomal dominant hereditary cerebral small vessel disease.

## Introduction

Cerebral small vessel disease (CSVD) is a set of heterogeneous diseases due to the pathological changes of cerebral arterioles, venules and capillaries (1–3). Stroke and cognitive impairment are the main clinical features of CSVD, which is mainly characterized by cerebral white matter lesions such as lacunar infarction and micro-hemorrhage in clinical imaging.

CSVD includes the sporadic and the familial hereditary cerebral small vessel disease. Sporadic CSVD is related to aging and hypertension, while the familial hereditary CSVD frequently starts early and can be inherited in an autosomal dominant inheritance manner (3–5). It has been confirmed that familial hereditary CSVD is related to certain types of mutation of a single gene. Cerebral autosomal dominant with subcortical infarcts and leukoencephalopathy (CADASIL) was associated with Notch3 mutation (6). Hereditary cerebral amyloid angiopathy is caused by COL4A1/COL4A2 mutation (7,8), and CARASIL by the high temperature requirement protease A1 *HTRA1* allele mutations, which is also called *HTRA1* homozygous mutations (9–13).

However, only very few patients have a clear gene mutation and the cause of the majority of familial cerebral small vessel disease cases is still unknown. To find out the probable gene mutation location, we analyzed a patient who was diagnosed as Notch3-negative but CADASIL-like (14). The hereditary cerebral vascular gene screening by direct sequencing and the single nucleotide polymorphism (SNP) of the candidate gene were analyzed by high-throughput multiplex polymerase chain reaction to find the causative gene of the family cerebrovascular disease.

## Material and Methods

### Patient

Clinical data was collected from a case of a suspected CADASIL patient and his family members in the Department of Neurology at the first hospital of Shanxi Medical University. The Notch3 gene consists of 33 exons, and we have examined 29 exons except the 4 exons 17/21/22/23, which do not belong to the hot spot mutations. The genetic diagnosis of Notch3 and skin biopsy of the patient were both negative. The patient and his family members who participated in the study all gave written informed consent.

According to our investigation, the pro-band did not have alopecia, spondylosis, and adult-onset Alexander's disease. Pedigree analysis was performed for the pro-band and his family, and there were two major features: 1) stroke and progressive cognitive impairment as the major clinical characteristics; 2) negative Notch3. Stroke is an ischemic or infarct injury of the brain that results in neurological deficit and contralateral impairments in verbal, cognition, motor and sensory system. Stroke and cognitive impairment are inter-related.

### Blood samples

Blood samples of the pro-band were collected and used for screening the hereditary cerebrovascular disease related genes, such as *HTRA1, APP, CST3, and ITM2B*, etc., and SNP genotyping assay were then conducted on family members after identifying the candidate gene.

### Genetic sequencing

The genetic sequencing of the 27 related genes of hereditary cerebrovascular disease were conducted. The 27 genes included *NOTCH3, HTRA1, KRIT1 (CCM1), CCM2, PDCD10 (CCM3), GLA, HBB, CBS, MMADHC, MTHFR, MTR, MTRR, PRKAR1A, F5, APP, CST3, ITM2B, ENG, ACVRL1, SMAD4, ABCD1, ARSA PLP1, MLC1, ASPA, GFAP,* and *GALC*.

### SNP genotyping assay for the candidate gene

To extract DNA, EDTA was added to venous blood (2 mL) for anticoagulation, and the column method (QIAamp Blood DNA Mini Kit, Qiagen,USA) was used for extracting genomic DNA from peripheral blood leukocytes. DNA was stored at 4°C.

Gene sequences were obtained from the human genome database GenBank. The Primer Premier 5.0 software was applied to design primers of the *HTRA1* gene exon coding region, using 2 X PCR MasterMix polymerase (Tiangen) for PCR amplification (ABI9700 PCR Instruments, Life Technology, USA). The PCR products from the specific primers were direct sequenced by Taiyuan Jinyu Clinical Inspection Ltd. using ABI3500 (Life Technology, USA). Then, reference sequences (NM_002775.4 and NG_011554.1) were used for comparison to localize the possible gene mutation.

### Bioinformatics analysis

Sequencing results were analyzed by PolyPhen2 (Polymorphism Phenotyping v2, a tool that can predict the possible impact of an amino acid substitution on the structure and function of protein), SIFT (sorting intolerant from tolerant) and Mutation Taster to see whether there is a likelihood of pathogenesis by gene mutation. SIFT predicts whether an amino acid substitution can affect protein function and prediction is based on the degree of conservation of amino acid residues in sequence alignments derived from closely related sequences, collected through PSI-BLAST.

## Results

### Clinical data of the patient and his family members

**Figure 1.**
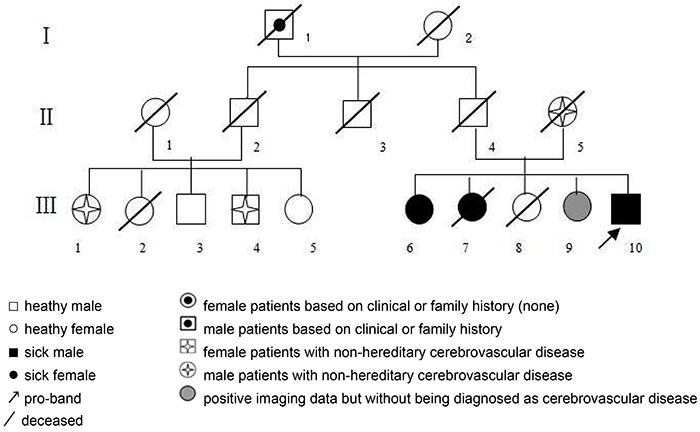
Pedigree chart of the pro-band.

Based on the pedigree chart of the pro-band (Figure 1), there was a clear family history of cerebrovascular disease on the paternal side, while the disease did not exist on the maternal side, inferring that the disease was originated from paternal autosomal dominant cerebrovascular disease. In the 17 people of the three generations, there were 6 with early-onset of cerebral vascular disease except the pro-band. Among the 6 people, there were 4 who had been considered genetically correlated for cerebral vascular disease, 3 (I1, II4, III7) were already deceased, and one (III6) was alive. Moreover, one person was considered “patient”, based on imaging diagnosis. II5, III1, and III4 had symptoms of cerebral vascular disease, but lacked imaging data, therefore they were not considered genetically related for the disease. The cause of death of II2 was unknown (might have been caused by cerebrovascular disease).

The pro-band, with onset of the disease at the age of 35 years, had no hypertension, diabetes, smoking or other risk factors of cerebrovascular disease, and there was no family history of cerebrovascular disease. In 2006, he presented with dizziness and double lower extremity weakness. In 2008, 2009, and 2011, he presented with similar symptoms, and double lower extremity weakness appeared alternately. After cerebral infarction treatment, the symptoms were slightly reduced. In 2012, he presented with dizziness and double lower limb weakness that were more serious, and required hospitalization. There was no definite risk factor for cerebrovascular disease. On physical examination, findings were clear mind, slower verbal reaction, slightly dull, the tongue to the right, limb muscle strength of grade 5, hypermyotonia, hyperreflexia, tendon hyperreflexia, bilateral symmetry for sense of depth, and bilateral Babinski syndrome (+). In cranial MRI, findings were multiple lacunar infarction in bilateral basal ganglia, lateral ventricles, and semi-oval center, and changes of bilateral periventricular white matter demyelination.

III6 was the sister of the pro-band, born in May 1955. In 2003, she was diagnosed with cerebral infarction for the first time, and it recurred once a year on average. In 2012, she presented with recurrent right limb numbness and weakness, and slurred speech. Symptoms aggravated in 4 days, and she required hospitalization. On physical examination, she was found to be clear-minded, with slurred speech, shallow right nasolabial fold, tongue to the right, positive right limb paresis test, and right Babinski syndrome (+). The imaging findings were similar to the pro-band.

**Figure 2.**
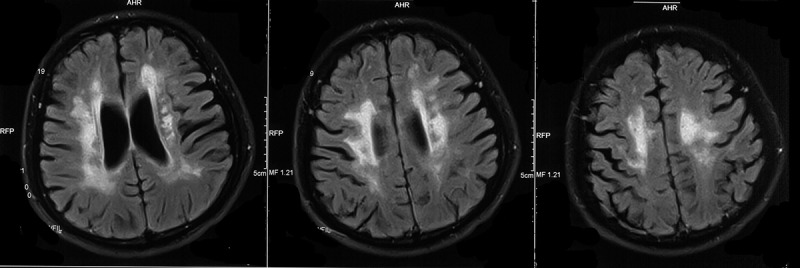
Head FLAIR and MRI images of the pro-band. Image shows bilateral lateral ventricles, bilateral semi-oval center with multiple abnormal signals, and demyelination in the white matter around the bilateral lateral ventricles.

Head MRI imaging data (Figures 2-4) suggested multiple lacunar infarction and diffuse cerebral white matter lesions. However, no obvious abnormality was observed from the cardiovascular-related examination (including 12 lead echocardiography, carotid artery ultrasound, transcranial Doppler, blood routine examination, and blood biochemical analysis). The diagnosis criteria of the CADASIL/CADASIL-like disease were satisfied by combining clinical symptoms, imaging data, and positive medical history. However, subsequent Notch3 gene test and skin biopsy excluded CADASIL diagnosis. Therefore, the disease was initially classified as CADASIL-like (15), or autosomal dominant hereditary cerebral small vessel disease without clear genetic diagnosis.

**Figure 3. f03:**
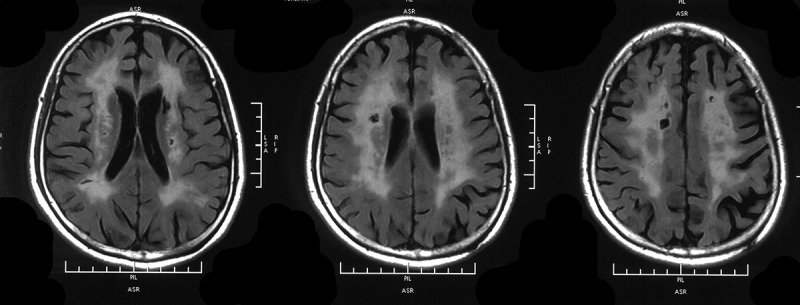
Head MRI and FLAIR images of III6. Image shows bilateral lateral ventricles, bilateral semi-oval center with multiple abnormal signals, and demyelination in the white matter around the bilateral lateral ventricles.

**Figure 4.**
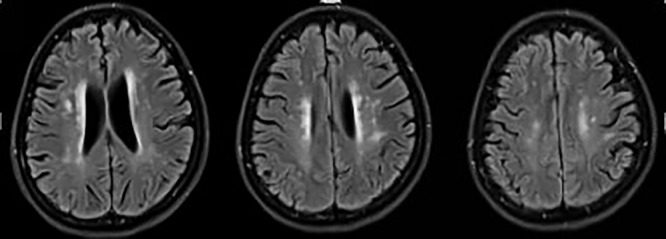
Head MRI and T2WI images of III9 (currently no clinical symptoms). Image shows bilateral lateral ventricles, bilateral semi-oval center with multiple patchy abnormal signal, and the white matter of the bilateral lateral ventricle slightly changed.

### Mini Mental State Examination (MMSE) score

The pro-band score was 17 on the MMSE, which was low considering that he was a graduate of technical school (normal would be a 24 score), indicating moderate damage of neurologic function. The III6 score was 22, considered low as she had a bachelor degree, indicating slight damage of neurologic function. The III9 score was 28, and his educational level was bachelor degree, indicating he was normal.

### Candidate genes and SNP test results

**Figure 5.**
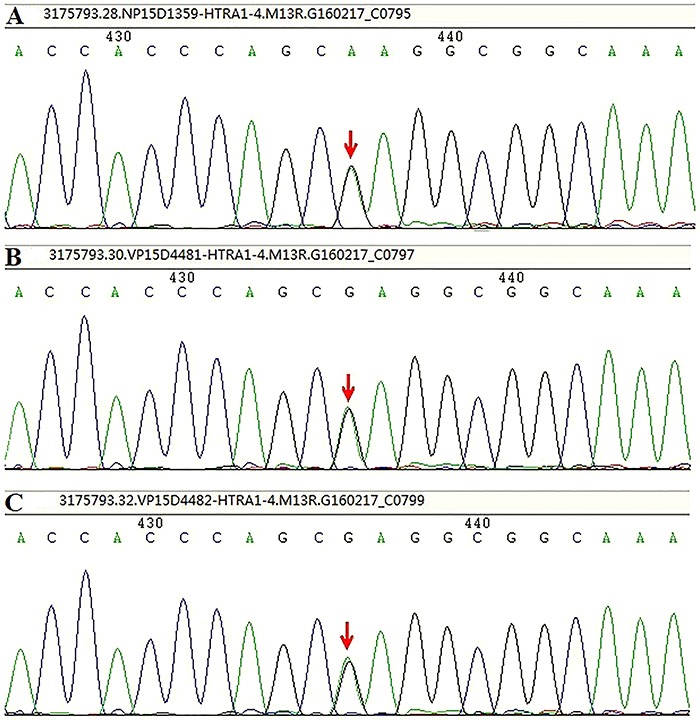
Single nucleotide polymorphism sequencing of candidate genes. Graphs show the c.905g>A Heterozygous mutation in of the *HTRA1* gene of the (*A*) pro-band (III10), (*B*) pro-band's eldest sister (III6) and (*C*) the pro-band's third sister (III9).

Direct sequencing was performed for the exons of hereditary cerebrovascular disease related genes from the pro-band, and the sequencing result was then compared with normal gene sequence. No significantly related gene mutations were found. However, a heterozygous missense mutation was detected in *HTRA1*, i.e., *HTRA1* (NM_002775.4) Exon4 c.905G>A p. (Arg302Gln). The biological software analysis with PolyPhen-2, SIFT, and Mutation Taster suggested that this missense mutation very likely caused cerebrovascular disease. Subsequent SNP genotyping assay of the candidate gene in III6 and III9 indicated that the *HTRA1* gene had the same single nucleotide polymorphism, strongly suggesting that this heterozygous missense mutation is the reason for family disease (Figure 5).

## Discussion

In this study, a CADASIL-like patient and his family members were investigated. The candidate gene heterozygous *HTRA1* missense mutation was obtained by genetically screening the currently known genes and hereditary cerebrovascular disease related genes. Then, the SNP genotyping assay of the candidate gene was performed for the family members to obtain the preliminary results.

The *HTRA1* gene is located at the long arm of chromosome 10 (10q26) encoding a series of serine protease and its encoding product is widely expressed *in vivo* (15). This protein family is composed of four domains from N-terminus to C-terminus, including insulin-like growth factor binding domain, Kazal domain, trypsin-like enzyme peptide domain, and PDZ domain. *HTRA1* is a gene that encodes a serine protein, which can regulate insulin-like growth factor (IGF) by cleaving the IGR binding protein. In addition, *HTRA1* was reported to regulate cell proliferation activity (16).

It is commonly recognized that the CARASIL disease is caused by the mutation of the *HTRA1* allele, whereas heterozygous mutations are not pathogenic (17). However, a recent study reported that HTRA1 heterozygous mutation could cause autosomal dominant cerebral small vessel disease (18). The conclusion was drawn by selecting candidate genes via gene sequencing, and then the genome comparison was carried out in a relatively large sample of a specific population. The study also indicated that the mutation might account for a large proportion of the non-CADASIL autosomal dominant cerebral small vessel disease cases. The genetic pattern of the disease may be attributed to the change in the content and activity of the encoded product due to gene mutation, rather than the gene itself.

The current study found that heterozygous *HTRA1* missense could lead to the arginine to glutamine change for the encoded protein located at 302nd amino acids. The mutation mode leads to a change in the structure of the protein, which may lead to changes in the spatial structure, and then to the change of the protein physical and chemical properties, physiological characteristics etc. Differently than HGMD, ESP6500, and dbSNP databases, this mutation has not been included in databases. Therefore, the SNP of *HTRA1* is considered not present in the normal population, but present in the family members who are either diagnosed with cerebrovascular disease or positive in clinical imaging. The clinical imaging of subject III6 indicated the presence of disease but there was no clear clinical symptoms yet, which still needs further follow-ups. This result suggested that the genetic missense mutation was first observed in clinical imaging, and then in clinical manifestations. At the same time, we have found that onset of the disease occurred in younger family members, which complies with genetic characteristics of the early occurrence.

In summary, the heterozygous *HTRA1* mutation is the cause of the disease, and the effects of the single gene can be enhanced in the genetic process. However, further research is needed to understand the physicochemical properties and biological activity of the encoding protein caused by gene mutation. In addition, it is not clear whether the SNP is present in the normal population. Lastly, we selectively performed partial gene screening of the pro-band rather than the entire sequencing, so whether the gene mutation acts individually or in combination with other types of mutation for the family members is unknown and needs further investigation.
